# Laparoscopic Surgery in a Patient With Endometrial Cancer After Laparoscopic Sacrocolpopexy

**DOI:** 10.7759/cureus.92205

**Published:** 2025-09-13

**Authors:** Kazunobu Yagi, Tomoko Kuwata, Hiromi Kashihara, Chikako Kato, Masami Takeyama

**Affiliations:** 1 Urogynecology, Daiichi Towakai Hospital, Takatsuki, JPN

**Keywords:** endometrial cancer, laparascopic surgery, laparoscopic sacrocolpopexy, pelvic organ prolapse, pelvic reconstruction

## Abstract

In patients undergoing laparoscopic sacrocolpopexy (LSC) for pelvic organ prolapse (POP) repairs, occult uterine endometrial cancer may be discovered later. However, its detection poses unique surgical and oncological challenges, particularly when a mesh is involved. This case report describes a laparoscopic pelvic reconstruction with trachelectomy and bilateral salpingo-oophorectomy for occult uterine endometrial cancer in resected specimens after LSC. A 51-year-old female with stage III uterine prolapse and cystocele presented at our center. She subsequently underwent LSC and a laparoscopic supracervical hysterectomy. The patient had no atypical genital bleeding. Cervical and endometrial cytology performed one year earlier showed no evidence of malignancy. However, pathological examination revealed stage IA, grade 1 endometrioid adenocarcinoma. The patient underwent trachelectomy, bilateral salpingo-oophorectomy, and laparoscopic surgery for reconstruction 41 days after the initial procedure. Part of the mesh was removed using a cervical stamp, while the remaining mesh was sutured together. Twelve months after the second surgery, no recurrence of uterine endometrial cancer or POP was observed. This case highlights the importance of preoperative screening for endometrial malignancy in POP surgery and demonstrates a feasible approach for oncologic surgery with selective mesh preservation.

## Introduction

In Japan, laparoscopic sacrocolpopexy (LSC) for pelvic organ prolapse (POP) has been widely performed in hospitals since it was covered by insurance in 2016. Additionally, as the population ages, the number of patients with POP increases, supporting the rising trend of LSC. However, the incidence of uterine cancer is increasing owing to aging and obesity [1]. Therefore, studies have reported uterine endometrial cancer in specimens from total or subtotal hysterectomies after LSC [2].

Currently, no clear guidelines exist for additional treatment in cases of incidentally diagnosed endometrial cancer. In this case report, endometrial cancer (stage IA) was diagnosed based on imaging tests performed after LSC and tissue pathology of the excised specimen. Subsequently, laparoscopic trachelectomy, bilateral salpingo-oophorectomy, and pelvic reconstruction were performed. At 6 months postoperatively, the patient showed no evidence of cancer recurrence or POP. This report presents a case in which pelvic floor reconstruction was performed during surgery for incidentally diagnosed uterine cancer after LSC.

## Case presentation

The patient was a 51-year-old female with a body mass index of 24.6 kg/m2 who had undergone vaginal delivery twice previously (G2P2). Initially, she was diagnosed with uterine prolapse 10 years ago, which gradually worsened over time. According to the POP-Q progression classification, the patient had stage III uterine prolapse and cystocele. The ring pessary slipped out immediately after its insertion. Based on these findings, LSC was indicated. Pathological examination of the resected uterine body revealed endometrioid adenocarcinoma (G1, less than 1/2 myometrial invasion, no lymph vascular invasion), with no cancer in the uterine corpus stump. Postoperative contrast-enhanced computed tomography (CT) showed no adjacent organs invasion, no distant metastasis, and no lymph vascular invasion, and serum tumor markers (cancer antigen 125,carbohydrate antigen 19-9, and carcinoembryonic antigen) were within normal limits (Table 1).

**Table 1:** Laboratory findings of the patient AST, aspartate aminotransferase; ALT, alanine aminotransferase; BUN, blood urea nitrogen; Cre, creatinine; BS, blood sugar; WBC, white blood cell; Hb, hemoglobin; Ht, hematocrit; Plt, platelet; CA125, cancer antigen 125; CA19-9, carbohydrate antigen 19-9; CEA, carcinoembryonic antigen

	Patient data	Normal range
Total protein (g/dL)	7.8	6.5 – 8.3
AST (U/L)	15	8 – 38
ALT (U/L)	12	4 – 43
BUN (mg/dL)	16	8 - 22
Cre (mg/dL)	0.7	0.47 – 0.79
BS (mg/dL)	118	60 – 109
WBC (/µL)	3800	3500 – 9100
Hb (g/dL)	11.9	11.3 – 15.2
Ht (%)	35.8	33.4 – 44.9
Plt (×10^4^ /µL)	15.1	13.0 – 36.9
CA125 (U/mL)	27.5	<35
CEA (ng/mL)	1.2	<5
CA19-9 (U/mL)	4	<37

Based on these findings, stage IA uterine cancer was suspected, and laparoscopic trachelectomy, bilateral oophorectomy, and mesh refixation were performed 41 days after the LSC.

Laparoscopic surgical technique

**Figure 1:**
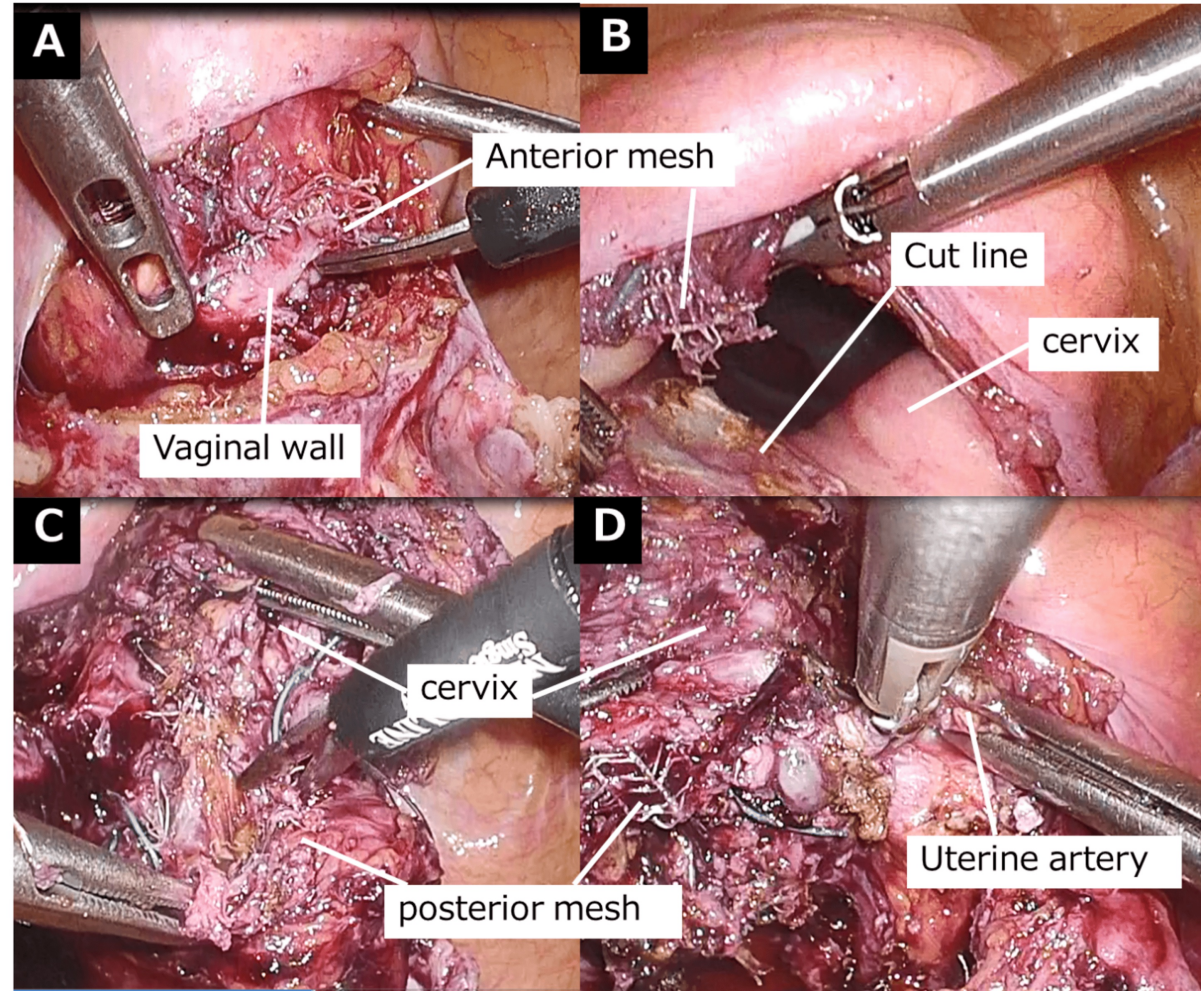
Laparoscopic trachelectomy (A) Incision of the anterior vaginal wall mesh. (B) Marking of the incision line. (C) Incision of the posterior vaginal mesh. (D) Incision of the right vaginal wall.

The patient was placed in a 15° head-down position on the operating table. The insufflation pressure was set at 20 mmHg. Four laparoscopic ports were inserted in a diamond configuration to access the surgical site. The sigmoid colon was suspended over the left lateral abdominal wall to facilitate its visualization. The uterine cervix was covered by the peritoneum. The mesh was secured to the cervix using a double-mesh on the anterior and posterior walls. The uterine cervix was grasped transvaginally with forceps and pushed up toward the peritoneal cavity. The tip of the vaginal delineator was then inserted into the anterior vaginal fornix. The peritoneum was incised toward the tip of the vaginal delineator. The anterior wall mesh was incised at the vaginal fornix level (Figure 1A), followed by a vaginal wall incision. A circumferential vaginal mucosal incision was made to allow for cervical resection (Figure 1B). The posterior wall mesh was incised at the vaginal fornix level (Figure 1C), followed by a vaginal-wall incision. During the incision of the left and right vaginal walls, the ascending branches of the uterine arteries on both sides were cauterized first, with careful attention to the course of the ureters (Figure 1D).

**Figure 2:**
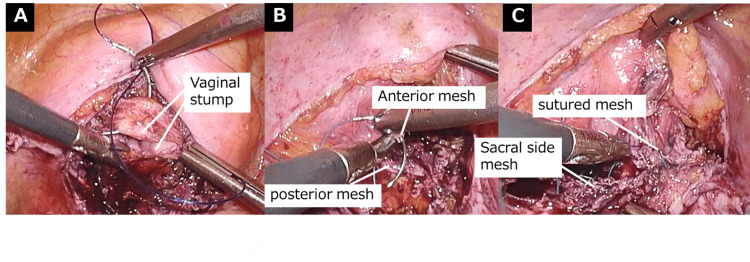
Laparoscopic reconstruction (A) Suturing of the vaginal stump. (B) Suturing of the anterior and posterior meshes. (C) Sacral-side and sutured (B) meshes.

The ovaries and uterine cervix were transvaginally removed and retrieved. The vaginal stump was closed with two continuous layers of sutures (Figure 2A). The anterior and posterior vaginal meshes were sutured to the vaginal wall using four single stitches (Figure 2B). The sutured and sacral-side meshes were sutured using four additional single sutures (Figure 2C).

Outcomes and follow-up

The operative duration was 134 min, with an estimated blood loss of 30 mL. No cancer cells were identified in the cervix, ovaries, or ascitic fluid. Therefore, the final diagnosis was stage IA2, G1 endometrial cancer [3]. Neither subjective nor objective recurrence was observed at 6 months follow-up. No preoperative and postoperative menstrual issues have been observed to date. Postoperative stress urinary incontinence (SUI) has been observed.

## Discussion

The prevalence of incidental uterine cancer after hysterectomy for POP is 0.6%, making it rare [4]. However, with an aging population, the incidence of POP and endometrial cancer complications is expected to increase. In POP surgeries involving meshes, LSC has replaced tension-free vaginal mesh for treating uterine prolapse with DeLancey level 1 injury, following the US Food and Drug Administration recommendations. Mourik et al. reported that LSC combined with subtotal hysterectomy has a lower recurrence rate of POP than LSC with uterine preservation [5]. Supracervical preserves sexual function and reduces mesh erosion better than total hysterectomy in LSC [6]. Therefore, suprahysterectomy is performed in many hospitals. However, uterine cancer is diagnosed in approximately 0.2% of patients following hysterectomy for POP [7].

As the number of LSC procedures increases, the likelihood of detecting incidental endometrial cancer postoperatively and requiring additional treatment is expected to increase. In addition, surgery for POP is performed using robot-assisted laparoscopic sacrocolpopexy(RSC)**, **but because there is no difference in treatment outcomes between LSC and RSC [8], our institution performed the procedure using LSC due to cost considerations. The American College of Obstetricians and Gynecologists reaffirmed its recommendation for routine endometrial assessment before supracervical hysterectomy, stating that “amputation of the uterine corpus in the abdominal approach and morcellation of the corpus in the laparoscopic approach require adequate preoperative assessment of the endometrial cavity to exclude neoplasm” [9].

Endometrial cytology has a sensitivity of 88.8% and specificity of 98.5% [10]; however, the false-negative rate is high, at 10-20% [11,12]. Therefore, even when preoperative cytology is negative, additional evaluation is performed using transvaginal ultrasound and pelvic magnetic resonance imaging, with endometrial biopsy performed when necessary. As a precaution at our hospital, the uterus is placed in a specimen retrieval bag, morcellated within the bag, and extracted through the umbilicus. However, since this case, we have adopted a preference for removing the uterus without morcellation when possible, as fragmentation may hinder the accurate pathological staging of endometrial cancer. In this case, the specimen removed during LSC showed localized endometrial cancer in less than half of the uterine myometrium, with negative resection margins. Additional CT imaging revealed no lymph nodes or distant metastases.

As no established guidelines exist for additional surgical procedures in such cases, the patient subsequently underwent laparoscopic trachelectomy, bilateral oophorectomy, and pelvic reconstruction. Although there was a risk of injury to surrounding organs due to mesh adhesion, the mesh used for LSC was made of polytetrafluoroethylene mesh ORIHIME🄬 (95g/m2, code 12B1X00005, Kono Seisakusho, Tokyo, Japan), a material associated with minimal inflammation [13]. Additionally, because the reoperation was performed soon after the initial LSC, we anticipated minimal mesh adhesion and deemed minimally invasive surgery feasible for the preoperatively determined cancer stage. Regarding removal of the mesh, since the endometrial cancer in the resected specimen was confined to less than half of the uterine myometrium, only a minimal portion of the mesh was removed, while the remaining mesh was retained to prevent POP recurrence.

The mesh from the sacrum to the vaginal stump was left intact. By suturing the remaining anterior and posterior vaginal wall mesh without attaching it to the vaginal stump, we reinforced DeLancey level Ⅱ connective tissue, preserving its potential to prevent cystocele and rectocele. Although no cases of malignant tumors directly metastasizing through the mesh have been reported, evidence suggests that artificial implants, such as meshes, may induce chronic inflammation, potentially promoting tumor implantation [14]. Therefore, during surgery for malignant tumors following LSC, mesh removal is recommended if the implant is in direct contact with the malignant tumor or in a comparable anatomical position. The decision to preserve the mesh must be contingent upon the confirmed absence of evident preoperative metastatic involvement of the uterine serosa, parametrial tissue, or adnexa.

Additionally, because the effects of radiation therapy on the mesh remain unclear, the patient should be closely monitored. Surgical treatment for POP improves the quality of life; however, endometrial cancer surgery should take priority because of its prognosis. Therefore, the primary objective is to complete tumor resection, although selective preservation strategies may help prevent POP recurrence. The Shull procedure is regarded as an appropriate surgical option for cases requiring mesh removal and is a common technique for vaginal stump fixation that maintains the anatomical and physiological position of the vaginal canal. It effectively restores the vaginal axis and preserves sexual function [15]. 

Although no postoperative urinary incontinence was observed in this case, it has been reported that urinary leakage can occur after surgery for POP [16,17]. It has also been reported that postoperative SUI is affected by sexual activity [18,19], so it is important to ask about the presence or absence of SUI as well as the recurrence of POP.

## Conclusions

In the presented case, endometrial cancer was diagnosed after LSC, prompting an additional laparoscopic surgery. This highlights the need for thorough preoperative screening for malignancies, particularly endometrial cancer. Although no recurrence has been observed, the 6-month follow-up period is short, and longer-term monitoring is necessary.
